# An integrative multi-platform analysis for discovering biomarkers of osteosarcoma

**DOI:** 10.1186/1471-2407-9-150

**Published:** 2009-05-16

**Authors:** Guodong Li, Wenjuan Zhang, Huazong Zeng, Lei Chen, Wenjing Wang, Jilong Liu, Zhiyu Zhang, Zhengdong Cai

**Affiliations:** 1 Department of Orthopaedics, Tenth People's Hospital, Tongji University, Shanghai 200072, PR China; 2 Department of Orthopaedics, Changhai Hospital, Second Military Medical University, Shanghai 200433, PR China; 3 School of Life Sciences, Fudan University, Shanghai 200433, PR China; 4 Shanghai Sensichip Co Ltd, Shanghai 200433, PR China; 5 International Co-operation Laboratory on Signal Transduction, Eastern Hepatobiliary Surgery Institute, Second Military Medical University, Shanghai 200438, PR China; 6 Shanghai Municipal Center for Disease Control & Prevention, Shanghai 200336, PR China

## Abstract

**Background:**

SELDI-TOF-MS (Surface Enhanced Laser Desorption/Ionization-Time of Flight-Mass Spectrometry) has become an attractive approach for cancer biomarker discovery due to its ability to resolve low mass proteins and high-throughput capability. However, the analytes from mass spectrometry are described only by their mass-to-charge ratio (*m*/*z*) values without further identification and annotation. To discover potential biomarkers for early diagnosis of osteosarcoma, we designed an integrative workflow combining data sets from both SELDI-TOF-MS and gene microarray analysis.

**Methods:**

After extracting the information for potential biomarkers from SELDI data and microarray analysis, their associations were further inferred by link-test to identify biomarkers that could likely be used for diagnosis. Immuno-blot analysis was then performed to examine whether the expression of the putative biomarkers were indeed altered in serum from patients with osteosarcoma.

**Results:**

Six differentially expressed protein peaks with strong statistical significances were detected by SELDI-TOF-MS. Four of the proteins were up-regulated and two of them were down-regulated. Microarray analysis showed that, compared with an osteoblastic cell line, the expression of 653 genes was changed more than 2 folds in three osteosarcoma cell lines. While expression of 310 genes was increased, expression of the other 343 genes was decreased. The two sets of biomarkers candidates were combined by the link-test statistics, indicating that 13 genes were potential biomarkers for early diagnosis of osteosarcoma. Among these genes, cytochrome c1 (CYC-1) was selected for further experimental validation.

**Conclusion:**

Link-test on datasets from both SELDI-TOF-MS and microarray high-throughput analysis can accelerate the identification of tumor biomarkers. The result confirmed that CYC-1 may be a promising biomarker for early diagnosis of osteosarcoma.

## Background

Biomarkers usually refer to specific genes and their products with biochemical features that can be used to measure the progress of disease or the effects of treatment. Both research scientists and clinicians are working towards integrating advanced high-throughput detection technologies, bioinformatics analysis tools, and biological experiments to discover and validate new biomarkers. Mass spectrometry and microarray technologies, commonly used in proteomic and genomic studies respectively, have become complementary tools for the discovery of biomarkers to build diagnostic models [1]. In microarray gene expression analysis, a candidate biomarker is usually a specific gene that is sequenced and annotated. Recently, SELDI-TOF-MS (Surface Enhanced Laser Desorption/Ionization-Time of Flight-Mass Spectrometry) technology, which has a high-throughput capability and can resolve low mass proteins, has rapidly developed as an attractive approach for cancer biomarker discovery from tissues or biological fluids. This technology combines mass spectrometry and chromatography to facilitate the characterization of protein profiles of complex biological mixtures. However, unlike probes on a microarray, the analytes from mass spectrometry are described only by their mass-to-charge ratio (*m*/*z*) values, without further identification and annotation. The result from mass spectrometry usually does not correspond to a specific intact protein; rather, it is a set of possible peptides that happen to be of the same mass or within a certain region.

Early tumor detection is a key to ensure effective treatment. Osteosarcoma (OS) is the most common primary high-grade bone tumor both in adolescents and in children [2,3]. Current standard treatment includes surgery based on the Enneking staging system and chemotherapy. For lack of early specific symptoms, the tumor has already developed into stage II_B _or III by the time it is confirmed in most patients [4]. Although some progress has been made over the years, the difficulties in early diagnosis are largely responsible for the unsuccessful treatment in 30–40% of patients with localized tumors and in 80–85% of patients with metastatic OS [5-7].

Applications of proteomic analyses have showed that amyloidrelated serum protein (SAA) might be utilized as a biomarker for OS. Li *et al*. used SELDI-TOF-MS to perform proteomic profiling on plasma specimens from 29 patients with OS and 20 age-matched patients with osteochondroma. The increased plasma level of SAA in OS patients was further validated by Western blotting [8]. Jin *et al*. implemented 2-D Fluorescence Difference Gel Electrophoresis (2-D DIGE) and MALDI-TOF-MS to analyze the serum proteins in patients with OS. The increase of SAA in serum was also confirmed by western blotting and ELISA in this study [5].

However, there are still technological limitations in cancer biomarker discovery. For example, several studies showed inconsistent biomarker sets for prostate cancer [9-11]. Lack of confirmation in various patients also poses a huge problem in the clinical application of mass spectrometry and microarray data to identifying disease-specific biomarkers [12,13]. A potential strategy to solve the problem is to consider and combine the consistent results from multiple platforms, including proteomics and genomics analysis. Deng *et al*. reported a new statistics algorithm named link-test to screen prostate cancer biomarkers from both microarray and SELDI mass spectrometry data sets [1]. The level of significance of the association between a microarray marker and a specific mass spectrum marker was determined by introducing background mass spectra distributions estimated by all human protein sequences in the SWISS-PROT database.

Cross-validation results showed high prediction accuracy using the identified biomarkers. The advantage of the multiple platform analyses is that the candidate biomarkers are identified by both mRNAs and protein products that reflect the expression levels of genes.

The purpose of this study was to prove the principle that it was possible to use the link-test to combine data from SELDI-TOF-MS and gene microarray analysis for biomarker discovery. Using this method, we identified a number of potential biomarkers for OS and further confirmed our findings by analyzing serum samples from patients with OS.

## Methods

### Specimens

The serum samples for SELDI-TOF mass spectrometry analysis were collected from 27 OS patients (age 8 – 26 years) prior to chemotherapeutics. The patients were from Shanghai Changhai Hospital, Ruijin Hospital, and Shanghai Sixth People's Hospital. For some patients, serum samples were collected before and after surgical operation. For each patient, 10 ml peripheral blood from the cubital vein was collected into an axenic, dry, and additive-free tube. The samples were centrifuged at 4,000 rcf for 10 minutes after being allowed to coagulate at 4°C for 4 hours to separate serum. Serum samples were stored at -80°C. Before analysis, serum samples were thawn on ice, and centrifuged at 4°C, 12,000 rcf for 10 minutes. At the same time, 47 serum samples of healthy individuals (age 7 – 29 years) were also collected as a normal control for SELDI-TOF-MS detection. All the healthy controls were the volunteers from a routine health examination. There were no significant age- or sex- differences between these two groups. All the serum samples were collected after informed consent had been signed. The studies were performed according to the rules of the Medical Ethics Committee of Second Military Medical University and Tongji University and approved by the local institutional review boards of participating institutions.

Three OS cell lines (MG-63, Saos-2 and U-2 OS) and one osteoblastic cell line (hFOB1.19) were used for gene microarray analysis. MG-63 was kindly provided by Dr Agi Grigoriadis (University College, London, UK). Saos-2, U-2 OS, and hFOB1.19 were purchased from ATCC (Manassas, VA, USA). All the cell lines were cultured with ATCC complete growth medium under the corresponding guidelines of ATCC standard conditions. The base medium for hFOB1.19 was a 1:1 mixture of Ham's F12 Medium Dulbecco's Modified Eagle's Medium, with 2.5 mM L-glutamine (without phenol red). To make the complete growth medium, add the following components to the base medium: 0.3 mg/ml G418, fetal bovine serum to a final concentration of 10%. The temperature for culture was 34.0°C. The complete growth medium for MG-63 was ATCC-formulated Eagle's Minimum Essential Medium, supplemented with 10% heat-inactivated fetal bovine serum. This cell line was cultured with 95% air and 5% carbon dioxide (CO_2_) at 37.0°C. The base medium forU-2 OS was ATCC-formulated McCoy's 5a Medium Modified. Fetal bovine serum was added into the base medium to a final concentration of 10%. The temperature for culture was 37.0°C. The base medium forSaos-2 was also ATCC-formulated Mc Coy's 5a Medium Modified. However, fetal bovine serum was added into the base medium to a final concentration of 15%. And this cell line was cultured with 95% air and 5% carbon dioxide (CO_2_) at 37.0°C. In order to meet the requirement of Affymetrix gene microarray hybridization (i.e. at least 1 μg total RNA can be extracted), all the cell lines were cultured at least for three generations until the total cell amount was more than 10^7^. All cells used were studied in exponential phase of growth and harvested when confluence of monolayer was observed.

### SELDI-TOF-MS analysis

Serum samples were analyzed by SELDI-TOF mass spectrometry using the CM10 protein chip of Ciphergen (Fremont, CA, USA). Chips were from one single batch. The serum was in no way fractionated prior to application to the ProteinChip surface. All the sera were run in one batch while pooled normal sera were used as control. The processing performed manually. 0.5 μl saturated solution of SPA in 50% CAN and 0.15% TFA was applied. The version of the mass spectrometer was Ciphergen ProteinChip Reader PBSIIc. The parameters of protein chip reader were set as follows: laser intensity was 150, detection sensitivity was 7, *m*/*z *values range was from 0 to 20,000, optimization range was from 2000 to 20000, and maximum molecular weight was 50000 Da. The instrument was normalized, and the analysis procedures were run in duplicate for twice. For each sample, there were two points. Each point was collected for 130 times. Data was processed using the Ciphergen ProteinChip Software 3.1.1. Spectra were normalized, calibrated and aligned.

Since the numbers of features were too large to build a reliable diagnostic model, peak detection was used to reduce the number of features. Peaks were basically the features with local maximum intensities. The obtained data were analyzed by Biomarker Wizard software to screen serum proteome biomarkers for OS. The effective protein peak was judged by S/N>5 and appearance frequency >10% in all original results. The statistical significance of peak height difference between samples from OS patients and those from healthy individuals was calculated by 2-sample t-test. To minimize false discovery rate, the Benjamini and Hochberg multiple test correction [14] was used. Peaks with an adjusted *p *value less than or equal to 0.05 were considered differentially expressed and were selected for further analysis. Hierarchical clustering of significantly changed peaks was conducted with MATLAB 7.5 (Mathworks, Natick, MA, USA) using default parameters (Euclidean distance metric and average linkage method).

### Gene microarray data

Total RNA of three OS cell lines and one osteoblastic cell line were extracted using the RNeasy Total RNA Isolation kit (Qiagen, Shanghai, China). The quality and purity of the products were assessed by an Agilent 2100 bioanalyzer (Agilent, Santa Clara, CA, USA). The criteria for RNA QC was RIN > = 7 and 28S/18S > = 0.7. Labeling was performed using the Affymetrix Genechip expression 3' amplification one-cycle target labeling and control reagent. Sample labeling and hybridization were performed according to standard procedures by Affymetrix systems. The 5 μg final synthesized biotinylated cDNAs were hybridized to Affymetrix GeneChip^® ^U133A arrays. Arrays were scanned with the Affymetrix scanner 3000. The transcript was considered as "expressed" only when the average intensity of signals in perfect match was 1.5 folds higher than that in mismatch, and the differences between the average intensity of signals were 4 folds higher than that of experimental noise.

Data analysis was performed using Microarray Suite 5.0 for each cell line compared to the osteoblastic cell line, respectively. Differentially expressed genes were selected using following criterion: 1) the average fold change between OS cell lines and osteoblastic cell line was more than or equal to 2 folds; and 2) the *p *value of single sample t-test was less than or equal to 0.05. T-test was conducted using MATLAB 7.5 (MathWorks, Natick, MA, USA). The genes were identified and annotated by online databases NetAffx™ Analysis Center http://www.affymetrix.com/analysis, GO http://www.geneontology.org, and GenBank http://www.ncbi.nlm.nih.gov/Genbank. Gene set enrichment analysis was performed using EASE software [15].

### Biomarkers identification

**Figure 1:**
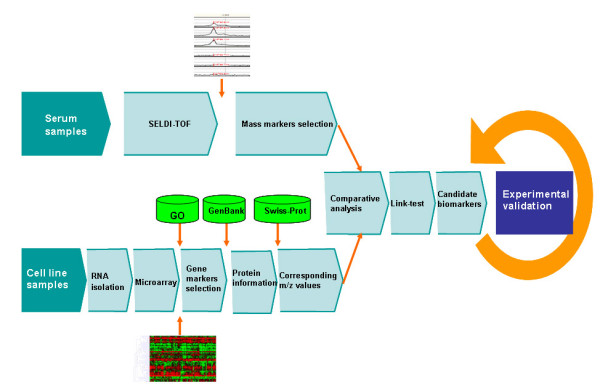
**Flowchart in this study**. Microarray and SELDI-TOF data were processed independently, and differentially expressed candidate markers were extracted from each type of analysis. Link tests were then applied to identify the significant common candidates from the association of microarray markers and mass spectrum markers. Finally, the candidate biomarkers were screened and expression of CYC-1 gene was validated by the experiments as an example.

We reasoned that the non-random peaks from mass spectrum analysis were likely to be originated from altered gene expression. Therefore, differentially expressed transcripts from microarray analysis were compared to the SELDI-TOF-MS data by link-test to determine the statistically significant hits [1]. Briefly, genes identified by microarray analysis were translated into corresponding proteins or peptides, and the expected *m*/*z *values were obtained from SWISS-PROT [16-19]http://www.expasy.ch/sprot/. Then, each *m*/*z *value of the peaks detected by SELDI-TOF mass spectrometry was compared with all those expected *m*/*z *values repeatedly. The significance of the result was evaluated by link-test. The *p *value was assigned to 0.05 by binomial test using default parameters with *δ *= 0.01[1]. The candidate biomarkers supported by both advanced high-throughput platforms were chosen for further experimental validation (see Figure 1). The algorithm of link-test was re-implemented in C/C++ code. All parameters were set by default.

### Immuno-blot analysis

Serum samples from eight OS patients and eight healthy individuals were subjected to SDS-PAGE, transferred to nitrocellulose, and blotted with the rabbit anti-human CYC-1 antibody, which was purchased from Protein Tech Group (Chicago, IL, USA). The bands were visualized with HRP-labeled goat anti-rabbit antibody and enhanced chemiluminescence (Pierce, Rockland, IL, USA). Protein concentration was determined using the BCA Protein Assay Kit (Pierce, Rockland, IL, USA). Equal amounts of proteins were loaded to each lane. After immuno-blotting, the expression levels of CYC-1 were normalized against the density of IgH, which was migrated around 50 kDa.

### Microarray data deposition

Microarray data in this study were submitted to NCBI GEO database http://www.ncbi.nlm.nih.gov/geo/ under the GEO number GSE14789 (including GSM369298, GSM369299, GSM369300 and GSM369301).

## Results

### Mass markers from SELDI-TOF-MS

**Figure 2:**
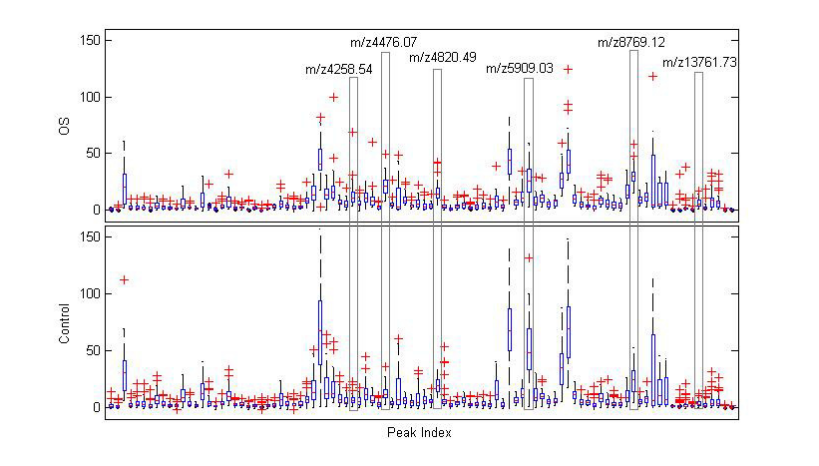
**Box Plots of the original mass markers exacted from SELDI-TOF mass spectrum analysis**. The abscissa was the m/z value of peak, and the coordinate was the log10 peak intensity. The six peaks that were statistically significant (*p *< 0.05) among the 96 differentially expressed protein peaks were marked.

**Figure 3:**
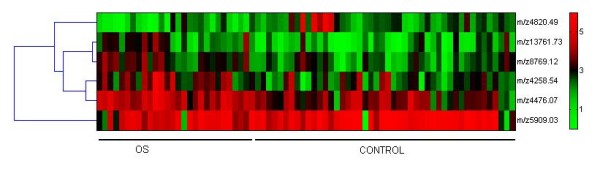
**Hierarchical cluster analysis of the significantly changed peaks**. Raw values were log2-transformed and centered relative to the median. Relative changes in their expression levels were indicated by a color code. Red indicated that the level of gene expression was higher than median, and green indicated that the level was lower than median.

The "case" dataset consists of 27 serum samples from OS patients, and the "control" dataset consists of 47 serum samples from healthy volunteers. The differences of fingerprint patterns between these two groups were analyzed using the Biomarker Wizard. After feature selection, an overall profile of peak intensities was presented as shown in Figure 2. 96 differentially expressed protein peaks were detected, which corresponded to proteins with molecular weight from 2,000 Da to 20,000 Da. Further statistical analysis indicated that six peaks of the "case' dataset were significantly different (*p *< 0.05) from corresponding peaks of the "control" dataset (Table 1). Among them, four peaks (*m*/*z *values were 4476.07, 8769.12, 13761.73 and 4258.54) represented up-regulation of corresponding proteins in samples from patients with OS, while the other two peaks (*m*/*z *values were 4820.49 and 5909.03) represented down-regulation of corresponding proteins in patients' serum. Hierarchical clustering analysis of these peaks was presented in Figure 3. Raw values were log2-transformed and centered relative to the median. Relative changes in their expression level were indicated by a color code. Red indicated that the level of gene expression was above the median, and green indicated that the level was below the median.

**Table 1:** Differentially expressed protein peaks between osteosarcoma patients and healthy control datasets (*p *< 0.05).

		Relative Peak Intensity			
			
Index	*m*/*z*	OS	CONTROL	OS/CONTROL	Adjusted *p *value
1	4258.54	13.93 ± 12.87	7.29 ± 4.84	1.91	3.57E-02
2	4476.07	22.11 ± 8.94	13.03 ± 6.80	1.70	6.79E-4
3	4820.49	3.20 ± 2.31	8.44 ± 10.53	0.38	2.45E-3
4	5909.03	28.21 ± 15.32	51.26 ± 28.65	0.55	3.14E-2
5	8769.12	9.32 ± 3.88	5.44 ± 2.79	1.71	2.83E-3
6	13761.73	6.98 ± 4.13	3.52 ± 2.74	1.98	1.12E-2

Relative peak intensities of Peak 1, 2, 5, and 6 were higher in OS case datasets than controls, and of Peak 3 and 4 were lower.

### Gene markers from microarray

Affymetrix^® ^HG-U133A microarray containing 14500 known genes (about 22000 transcripts) was used to compared the gene expression profile of osteoblastic cell line hFOB1.19 with that of three OS cell lines, MG-63, Saos-2, and U-2 OS. Compared with hFOB1.19 cells, the expressions of 968, 878, and 1150 genes were increased more than 2-fold in MG-63, Saos-2, and U-2 OS respectively. Meanwhile, the expressions of 888, 896, and 803 genes were decreased more than 2-fold in these three OS cell lines. 653 differentially expressed genes were finally selected as putative biomarkers based on the expression profiles and the following criterion: (1) the average fold change between the hFOB1.19 cells and the three osteoblastic cell lines was more than or equal to 2 folds; and 2) *p*-value of single sample t-test was less than or equal to 0.05, Among the 653 genes, 310 genes were potential up-regulated biomarkers, and 343 genes were potential down-regulated biomarkers (See Additional file 1 and 2).

**Figure 4:**
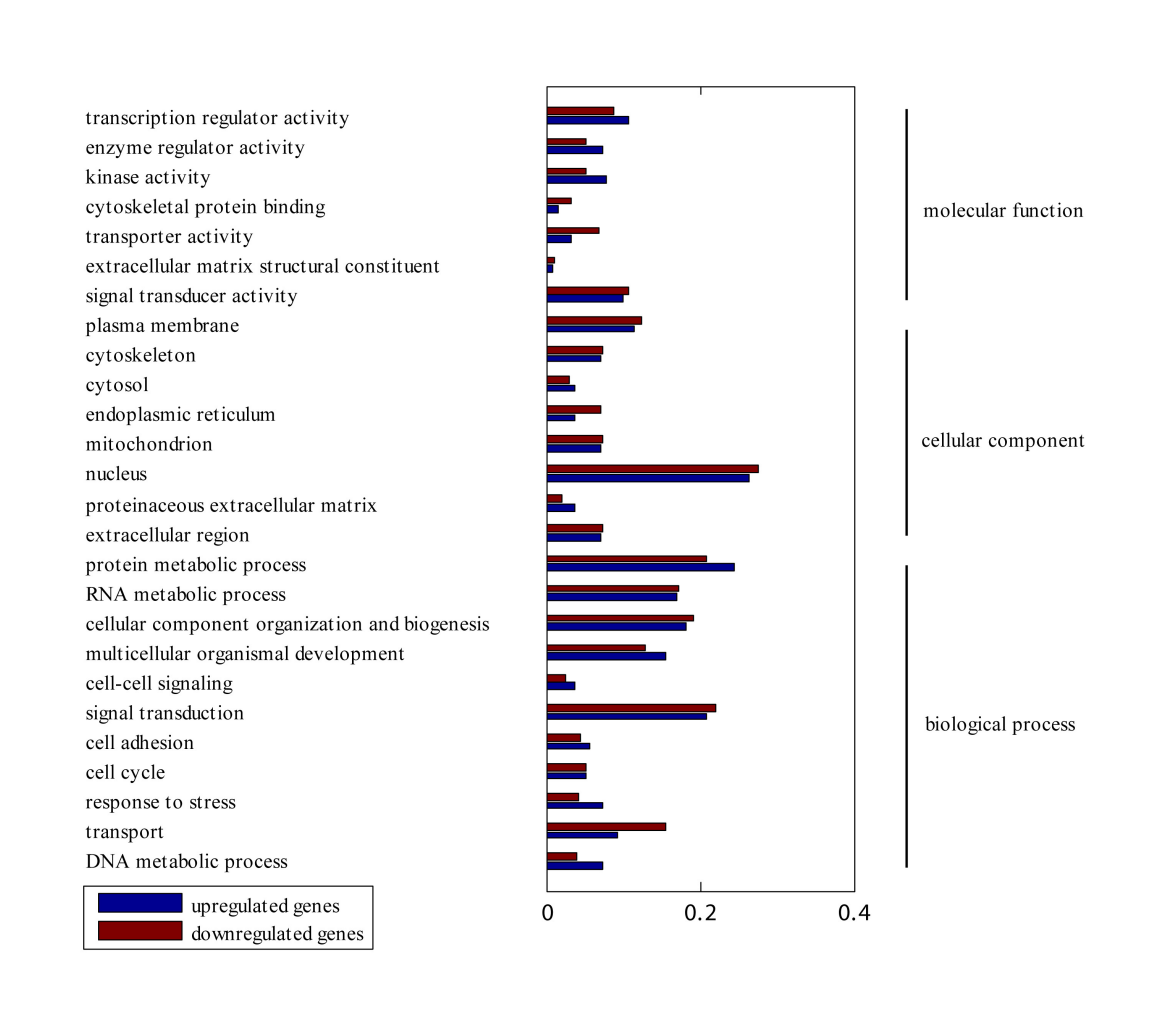
**GO analysis of biomarkers exacted from microarray analysis**. The abscissa of the Bar Plot was the proportion of genes within each Go category. All gene ontology terms listed on the bar plot had enrichment *p *values < 0.05. Unchanged genes annotated in the human genome were provided as background gene list.

Gene ontology analysis was performed by mapping each differentially expressed gene into records in the GO database http://www.geneontology.org/. The cluster analysis and GO annotation of these genes were presented in Figure 4. The abscissas of the bars represented the proportion of genes in the up-regulated and down-regulated genes list, respectively. All gene ontology terms listed on the graph has enrichment *p *values < 0.05.

### Biomarkers exaction from link-test

According to the mass spectrum markers and gene expression markers list, the candidate biomarkers for OS were identified by comparative analysis. The link-test determined 13 pairs of potential biomarkers with *p *value < 0.05. The results were summarized in Table 2. According to link-test, no association between microarray and SELDI data at m/z values of 4476.07 and 4820.49 was found, respectively. It suggested the corresponding biomarkers could not be considered as candidate proofs in this study because they were not supported by these two kinds of high-throughput screen methods.

**Table 2:** Candidate biomarkers from link-test.

*m*/*z*	Peak fold change (OS/CONTROL)	Microarray signal fold change (OS/CONTROL)	Link-test *p *Value	Gene_Swiss	Gene Symbol	Gene description
4258.54	1.91	2.94	0.018214	P51531	SMARCA2	SWI/SNF RELATED, MATRIX ASSOCIATED, ACTIN DEPENDENT REGULATOR OF CHROMATIN, SUBFAMILY A, MEMBER 2
4258.54	1.91	2.37	0.021632	Q9UIF9	BAZ2A	BROMODOMAIN ADJACENT TO ZINC FINGER DOMAIN, 2A
5909.03	0.55	0.38	0.042658	P40933	IL15	INTERLEUKIN 15
8769.12	1.71	2.28	0.024305	Q9H1D9	POLR3F	POLYMERASE (RNA) III (DNA DIRECTED) POLYPEPTIDE F, 39 KDA
8769.12	1.71	3.68	0.025301	P08574	CYC1	CYTOCHROME C-1
8769.12	1.71	2.44	0.026074	Q9UKF7	PITPNC1	PHOSPHATIDYLINOSITOL TRANSFER PROTEIN, CYTOPLASMIC 1
8769.12	1.71	2.68	0.027398	Q16342	PDCD2	PROGRAMMED CELL DEATH 2
8769.12	1.71	2.63	0.02806	Q9UBP4	DKK3	DICKKOPF HOMOLOG 3 (XENOPUS LAEVIS)
8769.12	1.71	5.2	0.028721	Q7LGA3	HS2ST1	HEPARAN SULFATE 2-O-SULFOTRANSFERASE 1
13761.73	1.98	2.31	0.012443	P15374	UCHL3	UBIQUITIN CARBOXYL-TERMINAL ESTERASE L3 (UBIQUITIN THIOLESTERASE)
13761.73	1.98	2.93	0.033783	O00115	DNASE2	DEOXYRIBONUCLEASE II, LYSOSOMAL
13761.73	1.98	2.3	0.043984	Q9GZL7	WDR12	WD REPEAT DOMAIN 12
13761.73	1.98	2.69	0.048002	Q9BT40	SKIP	SKELETAL MUSCLE AND KIDNEY ENRICHED INOSITOL PHOSPHATASE

### Experimental validation

**Figure 5:**
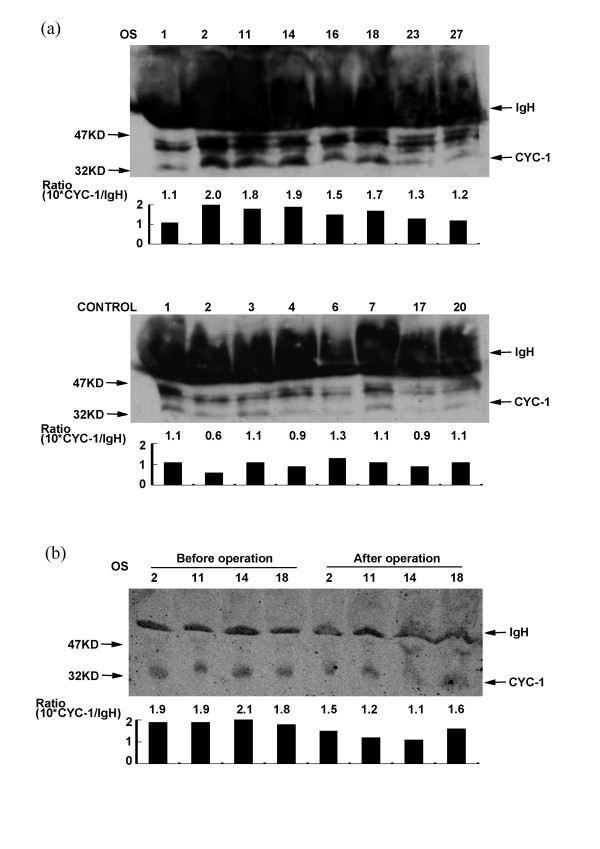
**(a) Expression of CYC-1 in the serum of osteosarcoma patients and healthy individuals**. (b) Expression of CYC-1 in serum samples of the same patient before and after surgery. The expression levels of samples were normalized according to the density scan of IgH (molecular weight was around 50 KD). Plot shown at the bottom panel represented 10 times of mean value of normalized CYC-1 intensity from three independent experiments.

To validate the specificity and sensitivity of the potential biomarkers in the early diagnosis, serum samples from eight patients with OS and eight healthy individuals were examined by immuno-blot analysis using antibodies specific for the selected proteins. For each western-blot analysis, three independent repeats were performed. The densities of specific bands from western-blots were scanned. After normalized with the density of immunoglobulin heavy chain (IgH), the values reflect the expression of these potential biomarkers in serum. The expression of the 35 kDa CYC-1 protein in serum from the 'case' and 'control' samples was shown in Figure 5a. The doublet above CYC-1 band was identified as non-specific band by test with irrespective antibodies like antibody of BCL-2. Plot shown at the bottom panel represents mean value of normalized CYC-1 intensity (× 10). The result indicated that the level of CYC-1 in serum samples from patients with OS was significantly higher than that from healthy controls (*p *< 0.05 by wilcoxon rank sum test). Furthermore, the levels of CYC-1 in serum samples of the same patient before and after surgery were compared. Significantly decreased expression was observed in patients 2, 11, and 14 (but not 18) as Figure 5b shown, indicating that CYC-1 may also be a useful biomarker for assessing the treatment of OS.

## Discussion

SELDI-TOF-MS Protein Chip and microarray analysis are widely used for identifying biomarkers for early diagnosis of cancer and for evaluation of treatment. A statistical test procedure named link-test has been built for detecting significance of biomarker candidates common to transcription and proteomics data sets. The identified putative biomarkers perform well in terms of prediction accuracy. They are supported by text mining in cross-databases and experimental results. The advantage of the methods applied in this study is that multiple platform analyses could improve the reliability of single measurements. In particular, the selection of the peak threshold in mass spectrum analysis is difficult and somewhat arbitrary, and the level of biomarker candidates is not fully quantitative. Link-test intends to overcome the problem of inconsistence in biomarkers discovery by providing a more refined candidate biomarker list for further research. Since the post-translational modifications could impact the links between the mRNA levels and the peaks in mass spectrum analysis, our method may be further improved by incorporating post-translational modification information from the SWISS-PROT database.

Although much progress has been made in the treatment of OS since 1970s, the five-year survival rate of OS patients has not been significantly improved in the last 10 years. Lack of early diagnosis is one of the most important reasons. In fact, over 90% of the patients already have suffered from tumors developed into stage II_B _or III when they are diagnosed [4]. Therefore, there is an urgent need to develop a rapid, specific and reliable method for the early diagnosis of OS. Since there are huge numbers of genetic and phenotypic changes in a given tumor cell, it is conceivable that genes or gene products involved in the pathogenesis of OS would serve as reliable markers for the tumor. To explore the pathogenesis-related genes, we developed an integrated early diagnosis workflow combining modern high-throughput detection approaches such as SELDI-TOF-MS technique and microarray expression analysis for identification of specific and sensitive biomarkers for OS. Ninety-six differentially expressed protein peaks were detected by SELDI-TOF-MS. Six of these were identified as potential serum proteome biomarkers with statistical significances. Meanwhile, 653 differentially expressed genes between OS cell lines and an osteoblastic cell line were identified by microarray analysis. The results from the three different OS cell lines were combined to reduce the influence of individual variation. Using the link-test statistical analysis, these two types of results were demonstrated to be associated, and yielded 13 genes considered as potential biomarkers of OS. Western-blot analysis of human CYC-1 confirmed the protein as one candidate biomarker in early diagnosis for OS.

Both plasma and serum contain various kinds of proteins. However, there is great difference for protein enrichment between plasma and serum. There is still no verdict of which one being more proper for detection. At present, most biomarkers of cancers are proteins in serum with low enrichments. Furthermore, there are limitations for dynamic range and sensitivity of instrument when discovering biomarkers in complicated samples such as plasma and serum. Some simple sample preparation method, like wipe off the proteins with highest enrichment, can help expanding the dynamic range of current instrument. In this study, in order to avoid the interference from fibrinogen with high enrichment, we selected serum samples to obtain more sensitive outcomes. It is worth noting that we used the CM10 chip, with a weak cation exchange surface, that is negatively charged for the mass spectrum analysis. Our results may skew toward potential markers that are positively charged at the experimental conditions. We are interested in analyzing the serum samples with Q10 chips to find more potential biomarkers.

Since the intrinsic characters of OS differ from other cancers such as liver cancer and prostate cancer, OS cell lines were chosen rather than peripheral blood mononuclear cells and tissue samples for gene microarray analysis. OS originates from stroma osteoblast in human bone marrow, and it is difficult to obtain the control corresponding to the OS tissue. Unlike the uniformity of cancer tissues such as liver and prostate cancers, the components of OS tissue are obviously influenced by sample location. The advantage of using standard cell lines is not only to get excellent control, but also reduce the bias by sampling. The surgically resected OS tissue has been also considered for gene microarray analysis in study. However, as the protein expression level of tumor cells could be different in vivo and in vitro, it still calls for exploration in the future studies. Moreover, application of the coincident result of differentially expressed gene list from three ATCC different osteosarcoma cell lines compared to osteoblastic cell line would help to reduce the influence of individual variation in this study.

CYC-1(cytochrome c1) is a heme-containing subunit of the cytochrome b-c1 complex that participates in the electron transfers electrons of the mitochondrial respiratory chain. The critical role of mitochondria in apoptosis has been demonstrated by numerous studies in various cells. For instance, Tafani *et al. *(2002) showed that CYC-1 was released upon Fas receptor activation [20]. A number of CYC-1 mutations related to tumor or cancer occurrence have been reported. In this study, we found that the levels of CYC-1 in serum samples from OS patients were higher than those from healthy controls. Furthermore, significant decrease of CYC-1 protein was observed in serum samples of 3 of 4 patients after surgical removal of the tumor. Further studies with a large pool of samples to test the reliability for more potential biomarkers are expected.

## Conclusion

Our results indicated that the integrative strategy combining the results from gene microarray, SELDI-TOF-MS and experiments could make it possible to identify potential biomarkers for early diagnosis of tumors or cancers expediently. In addition to CYC-1, we are currently verifying whether other potential biomarkers from the combined analysis are indeed associated with OS. Priority will be given to these genes related to cell growth and apoptosis.

## Competing interests

The authors declare that they have no competing interests.

## Authors' contributions

GL and WZ participated in the design of the multi-platform analysis, finished the biomarker selection and wrote the manuscript. WZ, HZ and JL provided the implementation of the algorithm for Link test. LC carried out the immuno-blot analysis. GL, WW and ZZ participated in the sample collection. ZC initiated the study and participated in its coordination. All authors have read and approved the final manuscript.

## Pre-publication history

The pre-publication history for this paper can be accessed here:

http://www.biomedcentral.com/1471-2407/9/150/prepub

## Supplementary Material

Additional file 1 **The cluster analysis of 653 differentially expressed genes**. The figure provided represents the cluster analysis of 653 differentially expressed genes. The logarithms of the expression level, from high to low, were denoted by color (from red, black, to green). The first line was the 'control' dataset, and the other lines were 'case' dataset from 3 OS cell lines MG-63, Saos-2 and U-2 OS. Click here for file

Additional file 2 **List of 653 differentially expressed genes**. The data provided shows the list of 653 differentially expressed genes. Click here for file
